# The Relationship Between Chronic Intermittent Hypoxia and MASLD and Fibrosis in Obstructive Sleep Apnea Patients

**DOI:** 10.3390/jcm15051911

**Published:** 2026-03-03

**Authors:** Sidem Gul, Songul Ozyurt, Caglayan Keklikkiran, Aziz Gumus

**Affiliations:** 1 School of Medicine, Recep Tayyip Erdoğan University Faculty of Medicine, 53100 Rize, Turkey; 2 Department of Pulmonary Medicine, Recep Tayyip Erdoğan University Faculty of Medicine, 53100 Rize, Turkey; azizgumus@gmail.com; 3 Department of Gastroenterology, Recep Tayyip Erdoğan University Faculty of Medicine, 53100 Rize, Turkey; c.keklikkiran@gmail.com

**Keywords:** fibrosis, liver, chronic intermittent hypoxia, MASLD, sleep apnea

## Abstract

**Background/Objectives**: Obstructive sleep apnea (OSA) causes recurrent apneas/hypopneas and intermittent oxygen desaturation during sleep. Chronic intermittent hypoxia (CIH) may be linked to metabolic dysfunction-associated steatotic liver disease (MASLD) and fibrosis through metabolic dysfunction. This study evaluated the relationship between OSA severity/hypoxemia indices and MASLD and fibrosis assessed by transient elastography. **Methods**: We prospectively enrolled 400 adults evaluated for suspected OSA at a respiratory disease outpatient clinic in Rize, Türkiye. All patients underwent overnight polysomnography. The apnea–hypopnea index (AHI), oxygen desaturation index (ODI), mean SpO_2_, and mean of each participant’s minimum SpO_2_ values were recorded. MASLD and fibrosis were assessed in the same individuals using FibroScan, with CAP (controlled attenuation parameter) and LSM (liver stiffness measurement) values recorded. OSA severity was categorized by AHI, and multivariable logistic regression was used to identify independent associations. **Results**: MASLD was present in 76% and fibrosis in 34.5% of patients. Patients with fibrosis had higher AHI (13.8 [8.2–35.2]) and ODI (11.5 [4.5–33.2]) and lower minimum SpO_2_ (*p* < 0.001). In multivariable models, BMI (OR 1.09; *p* < 0.001) and metabolic syndrome (OR 3.34; *p* < 0.001) were independently associated with MASLD, while BMI (OR 1.02; *p* < 0.001), metabolic syndrome (OR 2.03; *p* = 0.015), and ALT (OR 1.02; *p* = 0.032) were independently associated with fibrosis. **Conclusions**: MASLD and fibrosis were associated with OSA severity and hypoxemia before multivariable adjustment. However, after adjustment for obesity-related factors, liver outcomes were primarily explained by BMI and metabolic syndrome. Liver assessment should be considered in patients with OSA, particularly in those with high BMI and metabolic syndrome.

## 1. Introduction

Obstructive sleep apnea (OSA) is characterized by recurrent upper airway obstruction during sleep resulting in apneas/hypopneas and intermittent reductions in airflow [1]. It is more common especially in middle-aged and older individuals, and its frequency is increasing worldwide [2]. Although ethnicity, family history, alcohol and cigarette use, age and gender are among the risk factors of the disease, body mass index (BMI) is the most important risk factor [3], because obesity can impair respiratory mechanics, leading to a decrease in vital capacity and ventilation-perfusion imbalance. Additionally, snoring, increased respiratory effort, apnea, and hypopnea can cause repeated drops in oxygen saturation in the blood at regular intervals for a long time. This condition is defined as chronic intermittent hypoxia (CIH) and can adversely affect organ functions by triggering oxidative stress, inflammation, and sympathetic activation [4]. Obesity is also a key phenotypic risk factor for metabolic dysfunction-associated steatotic liver disease (MASLD), and evidence suggests that OSA and CIH may contribute to MASLD pathogenesis [5]. Experimental and clinical studies indicate that intermittent hypoxia can promote insulin resistance, altered hepatic lipid metabolism, MASLD, and fibrosis [6]. While OSA severity has been associated with fibrosis, CIH has been proposed as a potential mediator linking OSA to MASLD-related liver injury [7].

When the Oxygen Desaturation Index (ODI), which reflects intermittent hypoxia by measuring the frequency of oxygen desaturation per hour during sleep, is evaluated, it appears that lipid metabolism may contribute to both steatosis and fibrosis through the complex molecular pathway involving hypoxia-inducible factors (HIFs), inflammation, and fibrogenesis [8]. Several studies report higher rates of MASLD with increasing OSA severity [9]. In a study by Arısoy et al., steatosis rates increased across simple snoring and mild, moderate, and severe OSA (42.86%, 63.5%, 79.4%, and 79.2%, respectively) [10]. Similarly, Turkay et al. reported MASLD rates of 59%, 58.3%, and 78.2% in mild, moderate, and severe OSA, respectively [11]. Other studies have shown that MASLD severity is associated with apnea–hypopnea index (AHI) and ODI, and that minimum oxygen saturation (LSaO2) is lower in patients with MASLD [11,12]. Lower LSaO2 has also been reported in OSA patients with MASLD [13,14,15] Persistent inflammation in the presence of chronic hypoxia and oxidative stress may contribute to fibrosis progression in MASLD [6]. Consistently, studies have reported increased fibrosis risk in patients with coexisting OSA and MASLD [16,17]. These conditions support the hypothesis that there may be a relationship between OSA patients and fatty liver fibrosis. Therefore, this study aimed to evaluate the relationship between CIH and MASLD and fibrosis in patients evaluated for OSA in Rize, Türkiye.

## 2. Materials and Methods

This study is a prospective observational clinical study conducted at Recep Tayyip Erdoğan University Faculty of Medicine Training and Research Hospital between 30 May 2025, and 1 August 2025. Ethical approval was obtained from the Recep Tayyip Erdoğan University Non-Interventional Clinical Research Ethics Committee (15 May 2025; decision no. 2025/211; document no. E-40465587-050-1520). Written informed consent forms were obtained from the participants. OpenAI assistant was used solely to improve English language, grammar, and readability. The authors reviewed and edited the output and take full responsibility for the content of the manuscript.

Adult patients aged 18 years and older who presented to the respiratory diseases outpatient clinic and were suspected of having obstructive sleep apnea (OSA) were included in the study. All participants underwent a full-night video-assisted polysomnography (PSG, Natus® Brain Monitor PSG, Oakville, ON, Canada). overnight. Apnea was defined as events lasting at least 10 s and characterized by a ≥90% reduction in oronasal airflow. Hypopnea was defined as events lasting at least 10 s with a ≥30% decrease in airflow and a ≥3% desaturation or a ≥4% decrease in oxygen saturation. The Apnea–Hypopnea Index (AHI) was calculated by dividing the total number of apnea and hypopnea events of the participants by the total sleep time. Those with AHI < 5 were classified as simple snoring, those with 5 ≤ AHI < 15 as mild OSA, those with 15 ≤ AHI < 30 as moderate OSA, and those with AHI ≥ 30 as severe OSA. The oxygen desaturation index (ODI) was calculated as the number of desaturation events per hour of ≥3%. The minimum and mean oxygen saturation values of the patients were recorded.

Fasting venous blood samples were collected the morning after PSG. Laboratory measurements included glucose, total cholesterol, LDL cholesterol, HDL cholesterol, triglycerides, alanine aminotransferase (ALT), aspartate aminotransferase (AST), gamma-glutamyl transferase (GGT), albumin, and complete blood count. The Epworth Sleepiness Scale score was recorded, metabolic syndrome status was determined, and the Fibrosis-4 (FIB-4) index was calculated. All participants were evaluated for MASLD and fibrosis by an experienced gastroenterologist using transient elastography (FibroScan^®^ Expert 630, Echosens, Paris, France). The presence of fibrosis was accepted in patients with a Liver Stiffness Measurement (LSM) value of ≥6.0 kPa. The degree of fatty liver was assessed using the controlled attenuation parameter (CAP), and patients with a CAP of ≥238 dB/m were diagnosed with MASLD. MASLD was defined using CAP with a cut-off of ≥238 dB/m, and fibrosis was defined using LSM with a cut-off of ≥6.0 kPa, based on previously published validation studies/guidelines [18]. Measurements were performed using either the M or XL probe, selected according to the patient’s body mass index (BMI) and subcutaneous tissue thickness, in accordance with the manufacturer’s recommendations. The XL probe was used in patients with BMI ≥ 30 kg/m^2^, while the M probe was used in those with BMI < 30 kg/m^2^ [19]. In addition, skin and subcutaneous adipose tissue thickness were considered during probe selection to ensure optimal measurement accuracy. Reliability criteria included obtaining at least 10 valid measurements and an interquartile range to median ratio (IQR/median) ≤ 30%.

Individuals with alcohol-related liver disease were excluded using predefined thresholds of alcohol intake (e.g., >30 g/day for men and >20 g/day for women) and clinical history. Patients with viral hepatitis (HBV, HCV), liver malignancies, other known chronic liver diseases, history of hepatotoxic drug use, technically insufficient PSG data, and refusal to participate in the study were excluded from the study. Patients who met the inclusion criteria were evaluated for demographic and clinical data, such as age, gender, body mass index (BMI), waist and neck circumference, smoking, and comorbidities. Metabolic syndrome was defined according to the National Cholesterol Education Program—Adult Treatment Panel III ( NCEP ATP III version 2005) criteria as the presence of at least three of the following: waist circumference > 102 cm in men or >88 cm in women; triglycerides ≥ 150 mg/dL or lipid-lowering treatment for hypertriglyceridemia; HDL cholesterol < 40 mg/dL in men or <50 mg/dL in women or treatment for low HDL; blood pressure ≥ 130/85 mmHg or antihypertensive treatment; and fasting plasma glucose ≥ 100 mg/dL or treatment for elevated glucose [20]. Metabolic syndrome was included as a composite covariate representing overall metabolic risk burden. Hypertension was also included as an independent clinical comorbidity due to its established association with both OSA and liver disease.

Statistical evaluations are based on the IBM-SPSS program (SPSS version 21; SPSS Inc., Chicago, IL, USA). Normality of continuous variables was assessed using the Kolmogorov–Smirnov test. Continuous variables are presented as mean ± standard deviation or median (interquartile range), as appropriate, and categorical variables as number (%). In the comparison of the two groups, Student’s *t*-test was used for normally distributed variables and the Mann–Whitney U test was used for non-normally distributed variables. Categorical variables were compared with Chi-Square test. Univariable and multivariable logistic regression analysis was performed to determine the effect of anthropometric and OSA measurements on MASLD and fibrosis. The *p* < 0.05 value was considered statistically significant. Receiver operating characteristic (ROC) curves were constructed to assess the discriminative ability of OSA indices for MASLD, and sensitivity, specificity, positive predictive value, and negative predictive value were calculated.

## 3. Results

A total of 400 patients were included in the study. Of the participants, 42.5% were female (*n* = 170), and the mean age was 45.4 ± 11.7 years. Based on polysomnography, 32 patients (8.0%) were classified as simple snoring, 225 (56.3%) as mild OSA, 29 (7.2%) as moderate OSA, and 114 (28.5%) as severe OSA. A history of smoking was present in 39.5% (*n* = 158), and metabolic syndrome was identified in 44.0% (*n* = 176). Using CAP ≥ 238 dB/m, MASLD was detected in 304 patients (76.0%), and using LSM ≥ 6.0 kPa, liver fibrosis was detected in 138 patients (34.5%).

The demographic, clinical, polysomnographic, and laboratory characteristics of patients with and without MASLD are presented in Table 1a. Patients with steatosis were older (46.5 ± 11.3 vs. 42.1 ± 12.3 years) and more frequently male (61% vs. 46%). Metabolic syndrome (65% vs. 26%; *p* < 0.001) and hypertension (47% vs. 27%; *p* < 0.001) were more common in the MASLD group, and BMI as well as waist and neck circumferences were higher (all *p* < 0.001). In polysomnography, AHI and ODI were higher, while mean SpO_2_ and minimum SpO_2_ values were lower in patients with MASLD compared with those without MASLD (all *p* < 0.001). Laboratory analyses showed higher ALT, AST, GGT, CRP, triglycerides, and glucose, and lower HDL levels in the MASLD group (all *p* < 0.001). Univariable and multivariable logistic regression analyses were performed to identify factors associated with MASLD (Table 2a and Table 3a).

Fibrosis was detected in 138 patients (34.5%) based on LSM. Comparisons between patients with and without fibrosis are shown in Table 1b. The mean age was 46.9 ± 10.2 years in patients with fibrosis and 44.6 ± 12.3 years in those without fibrosis (*p* = 0.437). Metabolic syndrome and hypertension were more common in the fibrosis group (77% vs. 45%, *p* < 0.001; and 59% vs. 34%, *p* < 0.001, respectively). BMI was higher in patients with fibrosis (37.2 [32.0–41.6] vs. 30.8 [27.6–34.7]; *p* < 0.001), and waist circumference was also higher (118 ± 15 vs. 104 ± 17 cm; *p* < 0.001). AHI and ODI were higher in fibrosis cases, and the average nadir SpO_2_ (i.e., the mean of each participant’s minimum SpO_2_ value) was significantly lower (all *p* < 0.001). Mean SpO_2_ was also lower in the fibrosis group (*p* = 0.005). ALT, AST, and GGT were higher (all *p* < 0.001), while HDL was lower in the fibrosis group (*p* = 0.043).

In univariable analyses examining factors associated with fibrosis, hypertension, metabolic syndrome, BMI, AHI, ODI, minimum SpO_2_, CRP, ALT, and GGT were significant (Table 2b and Table 3b). In the multivariable model, BMI (OR = 1.019; *p* < 0.001), metabolic syndrome (OR = 2.034; *p* = 0.015), and ALT (OR = 1.019; *p* = 0.032) remained independently associated with fibrosis.

### ROC Curve Analyses

**Figure 1:**
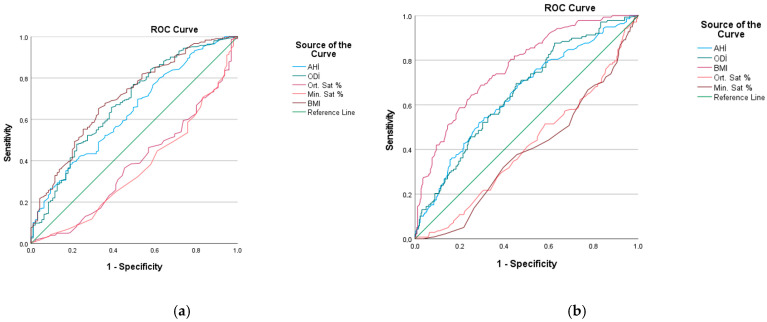
ROC curve analyses evaluating the discriminative performance of BMI and OSA-related parameters for liver outcomes. (**a**) MASLD (CAP-defined). (**b**) Liver fibrosis (LSM-defined).

ROC curve analyses were performed to evaluate the discriminative performance of selected measures for MASLD. The AUC values were 0.704 for BMI, 0.635 for AHI, and 0.676 for ODI (all *p* < 0.001), with BMI showing the highest discriminative ability (Figure 1a). Fibrosis stage distribution was as follows: F0, 65.5%; F1, 24.5%; F2, 5.5%; F3, 2.5%; and F4, 2.0%. For liver fibrosis, the AUC values were 0.769 for BMI, 0.647 for AHI, and 0.654 for ODI (all *p* < 0.001). ROC curve analyses for fibrosis are presented in Figure 1b.

## 4. Discussion

In this study, we investigated the relationship between OSA-related hypoxemia and MASLD and fibrosis in a relatively large cohort. We used hypoxemia indices as clinical markers of chronic intermittent hypoxia (CIH). We observed that MASLD (76%) and fibrosis (34.5%) were common among patients evaluated for OSA, and both were more frequent in those with more severe OSA and worse nocturnal oxygenation. However, after adjustment for BMI and metabolic syndrome, these associations weakened. This suggests that liver involvement in OSA is largely explained by obesity-related metabolic dysfunction.

Importantly, AHI and ODI, which reflect respiratory event burden and desaturation frequency, were higher in patients with MASLD and fibrosis, and minimum oxygen saturation values were lower in both groups. This pattern supports the clinical relevance of hypoxemia in the OSA–MASLD spectrum and is consistent with the concept that repeated desaturation and reoxygenation may contribute to metabolic dysregulation and inflammatory stress. At the same time, our multivariable findings emphasize that hypoxemia-related signals are closely intertwined with obesity and insulin resistance, making it difficult to separate “pure hypoxia effects” from the broader cardiometabolic phenotype in routine clinical cohorts.

Recent studies align with this overall framework. Jia et al. reported a linear relationship between OSA severity and MASLD among patients with type 2 diabetes [21], highlighting that the association may be particularly evident in metabolically vulnerable populations. Yu et al. further suggested that OSA may represent a causal risk factor for MASLD based on combined epidemiological and Mendelian randomization analyses [22]. However, biopsy-confirmed data have also shown that although OSA can be associated with advanced fibrosis, the association may lose significance after adjustment for obesity, underscoring the dominant role of adiposity-related confounding and/or mediation [23] Consistent with this, when we included BMI and metabolic syndrome in our multivariable models, the independent contribution of OSA indices diminished, supporting the interpretation that hypoxemia may act within a pathway largely shaped by obesity and insulin resistance rather than operating as a stand-alone determinant of liver injury.

From a biological standpoint, experimental evidence provides plausible mechanisms linking CIH to liver pathology. CIH has been reported to accelerate fibrogenesis through increased HIF-1α and TGF-β1 activation, and hypoxia–reoxygenation cycles can induce lipid peroxidation and NASH-like histological changes [24]. Related experimental work further demonstrates that oxidative stress and inflammation triggered by CIH can promote fibrogenic pathways [24,25]. In our study, patients with liver fibrosis had significantly higher levels of CRP, ALT, AST, and GGT, which may provide indirect clinical support for the proposed mechanisms linking chronic intermittent hypoxia (CIH) to liver injury. Elevated CRP reflects a state of systemic inflammation, which is a key mediator of hypoxia-induced hepatic injury. Similarly, increased levels of ALT, AST, and GGT suggest hepatocellular injury and oxidative stress, both of which are known consequences of CIH-related hypoxemia. These findings are consistent with experimental and clinical evidence indicating that CIH promotes hepatic inflammation, oxidative stress, and fibrogenesis. However, it should be noted that these biomarkers are not specific to hypoxia-induced liver injury and may also be influenced by other metabolic and inflammatory conditions.

Beyond environmental and metabolic influences, genetic susceptibility may also modify the OSA–MASLD relationship. Mendelian randomization data indicate that an OSA genetic risk score can increase the probability of MASLD, and this effect appears to be stronger in the presence of obesity [26]. Similarly, several studies suggest that the association between OSA and fibrosis is most evident in obese patients, pointing to effect modification by adiposity [27]. Taken together, these findings support a model in which OSA-related hypoxemia interacts with an obesity-driven metabolic background, amplifying MASLD and fibrogenic risk in susceptible phenotypes.

At the molecular level, Zhang et al. reported joint activation of NF-κB, TNF-α, and HIF-1 signaling pathways in both OSA and MASLD [28], which provides a coherent biological framework for the overlap between intermittent hypoxemia, inflammatory signaling, and hepatic lipid dysregulation. Additionally, a bidirectional relationship between sleep duration, oxygen desaturation, and MASLD has been reported [29], suggesting that sleep-related factors and hypoxemia may operate alongside metabolic stress exposure and underlying predisposition. Clinically, this reinforces the need to view OSA, obesity, and liver disease as interconnected components of a shared cardiometabolic continuum rather than isolated conditions. Clinical implications: From a clinical perspective, our findings suggest that liver assessment may be particularly relevant in patients evaluated for OSA who also have a high BMI and features of metabolic syndrome. Although hypoxemia indices were associated with MASLD and fibrosis in unadjusted analyses, these relationships were largely attenuated after accounting for obesity-related factors, indicating that risk stratification should prioritize overall cardiometabolic profile. Therefore, integrating metabolic risk assessment and considering noninvasive liver evaluation may help identify patients who could benefit from early lifestyle and metabolic interventions alongside OSA management. Also, we acknowledge that metabolic syndrome was included to reflect global metabolic risk rather than to investigate its individual contribution, which is beyond the primary scope of this study.

This study has several limitations. Firstly, because participants were recruited from a tertiary sleep clinic among individuals referred for suspected OSA, referral bias is likely. Such patients may differ from community-based OSA cohorts in symptom burden, disease severity distribution, and cardiometabolic comorbidity profile. Therefore, prevalence estimates (MASLD/fibrosis) and observed effect sizes may not be directly generalizable to the wider OSA population or to the general community, and external validation in multicenter and population-based cohorts is warranted. Second, the cross-sectional design captures associations at one time point and therefore cannot establish directionality or causality. Third, MASLD and fibrosis were assessed using transient elastography (CAP/LSM), which is practical for clinical studies but may not reflect histologic severity with the same precision as liver biopsy. In addition, CO_2_-related parameters (e.g., PetCO_2_ or PaCO_2_) were not routinely available; therefore, we could not evaluate the potential contribution of hypercapnia and respiratory acidosis to metabolic dysregulation or liver involvement in this cohort. Finally, although we adjusted for key metabolic factors, residual confounding from unmeasured variables cannot be ruled out.

## 5. Conclusions

In conclusion, MASLD and fibrosis were common among patients evaluated for suspected OSA. Although steatosis and fibrosis were more frequent in those with more severe OSA and worse nocturnal oxygenation, these associations were attenuated after adjustment for BMI and metabolic syndrome. Thus, the observed overlap between OSA and liver involvement appears to be largely related to obesity-associated metabolic dysfunction. Liver assessment may be considered in patients with OSA, particularly in those with high BMI and adverse metabolic features.

## Figures and Tables

**Table 1:** Clinical, anthropometric, polysomnographic, and laboratory characteristics of participants stratified by liver outcomes. (**a**) Comparison between patients with and without MASLD, defined by CAP. (**b**) Comparison between patients with and without hepatic fibrosis, defined by LSM. Data are presented as mean ± SD, median (IQR), or n (%), as appropriate.

**a.** Comparison of Cases with and Without MASLD				**b.** Comparison of Cases with and Without Hepatic Fibrosis			
	With MASLD (CAP ≥ 238)	Without MASLD (CAP < 238)	*p* value		With Hepatic Fibrosis (LSM ≥ 6)	Without Hepatic Fibrosis (LSM < 6)	*p* value
Number of patients (%)	304 (76)	96 (24)		Number of patients (%)	138 (34.5)	262 (65.5)	
Age (years)	46.5 ± 11.3	42.1 ± 12.3	<0.001	Age (years)	46.9 ± 10.2	44.6 ± 12.3	0.437
Gender, Male n (%)	186 (61)	44 (46)	0.008	Gender: Male n (%)	83 (60)	147 (56)	0.008
Smoker n (%)	121 (40)	37 (39)	0.826	Smoker n (%)	53 (38)	105 (40)	0.745
Hypertension n (%)	143 (47)	26 (27)	<0.001	Hypertension n (%)	81 (59)	88 (34)	<0.001
Metabolic Syndrome n (%)	199 (65)	25 (26)	<0.001	Metabolic Syndrome n (%)	106 (77)	118 (45)	<0.001
Height (cm)	170 (162–176)	167 (160–175)	0.084	Height (cm)	168 (162–175)	170 (161–176)	0.833
Weight (kg)	96 (85–10)	81.5 (72–91)	<0.001	Weight (kg)	105 (92–115)	87 (76–100)	<0.001
Waist circumference (cm)	112 ± 17	97 ± 16	<0.001	Waist circumference (cm)	118 ± 15	104 ± 17	<0.001
Neck circumference (cm)	41 (38–43)	38 (36–41)	<0.001	Neck circumference (cm)	42 (39.5–44)	39 (37–42)	<0.001
BMI (kg/m^2^)	33.7 (29.7–38.7)	29.4 (26.1–33.1)	<0.001	BMI (kg/m^2^)	37.2 (32–41.6)	30.8 (27.6–34.7)	<0.001
Epworth score	5 (2–8)	4 (2–7)	0.519	Epworth score	5 (2–8.5)	4 (1–7)	0.087
AHI	10.2 (7–33.9)	8.2 (6–11.9)	<0.001	AHI	13.8 (8.2–35.2)	8.7 (6.3–17.2)	<0.001
ODI	8.39 (3.44–24)	3.26 (0.63–9.27)	<0.001	Mean SpO_2_ (%)	93.4 (91.2–94.5)	93.9 (92.3–95)	0.005
Mean SpO_2_ (%)	93.4 (91.7–94.8)	94.1 (92.8–95.5)	<0.001	Minimum SpO_2_ (%)	84 (76–89)	87 (81–91)	<0.001
Minimum SpO_2_ (%)	85 (79–89.8)	89 (85–92)	<0.001	ODI	11.5 (4.5–33.2)	5.5 (1.7–14.9)	<0.001
CRP (mg/L)	3.46 (1.77–6.01)	2.24 (1.03–3.86)	<0.001	CRP (mg/L)	3.75 (2.09–7.26)	2.69 (1.34–5.00)	<0.001
WBC	7.16 (6.10–8.38)	7.03 (5.99–8.69)	0.573	WBC	7.14 (6.15–8.47)	7.15 (6.06–8.41)	0.495
ALT (U/L)	28 (20–39)	21 (16–29)	<0.001	ALT (U/L)	30 (22–44)	23 (17–33)	<0.001
AST (U/L)	22 (18–28)	20 (17–25)	0.012	AST (U/L)	24 (19–31)	20 (17–25)	<0.001
GGT (U/L)	28 (21–38)	21 (16–31)	<0.001	GGT (U/L)	31 (23–44)	24 (19–34)	<0.001
T. Bilirubin (mg/dL)	0.60 (0.46–0.77)	0.58 (0.43–0.79)	0.687	T. Bilirubin (mg/dL)	0.60 (0.46–0.78)	0.60 (0.45–0.77)	0.937
D. Bilirubin (mg/dL)	0.14 (0.11–0.08)	0.10 (0.07–0.13)	0.836	D. Bilirubin (mg/dL)	0.11 (0.07–0.14)	0.10 (0.08–0.14)	0.972
Glucose (mg/dL)	98 (90–109)	92 (86–98)	<0.001	Glucose (mg/dL)	100 (92–111)	94 (88–103)	<0.001
Total Cholesterol (mg/dL)	218 ± 49	211 ± 50	0.189	Total Cholesterol (mg/dL)	214 ± 42	218 ± 52	0.391
LDL Cholesterol (mg/dL)	138 ± 39	134 ± 42	0.430	LDL Cholesterol (mg/dL)	135 ± 34	138 ± 42	0.454
HDL Cholesterol (mg/dL)	44.9 (38.7–51.9)	50 (43.5–60.5)	<0.001	HDL Cholesterol (mg/dL)	44 (38.5–52)	47 (40–55)	0.043
Triglyceride (mg/dL)	160 (110–208)	109 (81–176)	<0.001	Triglyceride (mg/dL)	152 (109–211)	143 (97–197)	0.111

The data are given as mean ± standard deviation, median (IQR 25–75%) and n (%). **BMI**: Body mass index, **AHI**: Apnea–Hypopnea Index, **ODI**: Oxygen Desaturation Index, **Mean SpO_2_ (%)**: Mean peripheral oxygen saturation, **CRP**: C-reactive protein, **WBC**: White blood cell count, **ALT**: Alanine aminotransferase, **AST**: Aspartate aminotransferase, **GGT**: Gamma-glutamyl transferase, **T. bilirubin**: Total bilirubin, **D. bilirubin**: Direct bilirubin, **LDL cholesterol**: Low-density lipoprotein cholesterol, **HDL cholesterol**: High-density lipoprotein cholesterol.

**Table 2:** Univariable binary logistic regression analyses of factors associated with liver outcomes. (**a**) MASLD. (**b**) Hepatic fibrosis. Results are presented as odds ratios (ORs) with 95% confidence intervals (CIs) and *p*-values.

**a.** Demonstration of factors associated with MASLD by binary logistics univariable regression analysis			
	OR	%95 confidence interval	*p* value
Gender: Male	1.863	1.172–2.960	0.008
Hypertension	2.391	1.446–3.956	<0.001
Metabolic syndrome	5.382	3.221–8.995	<0.001
BMI	1.150	1.096–1.206	<0.001
AHI	1.031	1.013–1.049	<0.001
ODI	1.024	1.009–1.040	0.002
Min. SpO_2_	0.949	0.919–0.981	0.002
CRP	1.087	1.014–1.166	0.019
ALT	1.040	1.019–1.061	<0.001
GGT	1.033	1.014–1.053	<0.001
**b.** Demonstration of factors associated with hepatic fibrosis by binary logistic univariable regression analysis			
	OR	%95 confidence interval	*p* value
Gender: Male	1.181	0.776–1.795	0.438
Hypertension	2.810	1.837–4.297	<0.001
Metabolic Syndrome	4.042	2.541–6.431	<0.001
BMI	1.180	1.132–1.231	<0.001
AHI	1.027	1.015–1.039	<0.001
ODI	1.020	1.011–1.030	<0.001
Min. SpO_2_	0.963	0.942–0.986	0.001
CRP	1.061	1.012–1.112	0.015
ALT	1.027	1.014–1.041	<0.001
GGT	1.021	1.009–1.034	<0.001

**Table 3:** Multivariable binary logistic regression analyses of factors independently associated with liver outcomes. (**a**) MASLD. (**b**) Hepatic fibrosis. Results are presented as adjusted odds ratios (aORs) with 95% confidence intervals (CIs) and *p*-values.

**a.** Demonstration of factors associated with MASLD by binary logistics multivariable regression analysis			**b**. Demonstration of factors associated with hepatic fibrosis by binary logistic multivariable regression analysis			
	OR	95% confidence interval		OR	%95 confidence interval	*p* value
Hypertension	1.031	0.555–1.914	HT	1.957	1.192–3.211	0.008
BMI	1.093	1.037–1.152	BMI	1.170	1.117–1.226	**<0.001**
AHI	1.012	0.983–1.042	AHI	1.016	0.992–1.041	0.203
ODI	0.994	0.970–1.020	ODI	0.998	0.997–1.020	0.867
Min. SpO_2_	1.000	0.960–1.042	Min. SpO_2_	1.018	0.982–1.057	0.333
CRP	1.020	0.954–1.092	CRP	0.983	0.926–1.042	0.561
ALT	1.023	0.999–1.048	ALT	1.020	1.002–1.038	**0.030**
GGT	1.005	0.983–1.027	GGT	1.006	0.989–1.023	0.526

## Data Availability

The data presented in this study are available on request from the corresponding author. The data are not publicly available due to ethical and privacy restrictions.
